# Influence of HLA mismatch between donors and recipients on postoperative outcomes in cadaveric lung transplantation

**DOI:** 10.1007/s11748-024-02109-8

**Published:** 2024-12-09

**Authors:** Hidenao Kayawake, Ichiro Sakanoue, Satona Tanaka, Yojiro Yutaka, Yoshihiro Nishino, Akira Matsumoto, Taiki Ryo, Taichi Matsubara, Daisuke Nakajima, Hiroshi Date

**Affiliations:** Department of Thoracic Surgery, Kyoto University, 54 Shogoin-Kawahara-cho, Sakyo-ku, Kyoto, 606-8507 Japan

**Keywords:** Cadaveric lung transplantation, HLA mismatch, De novo donor-specific antibody, Chronic lung allograft dysfunction

## Abstract

**Objectives:**

Generally, HLA matching between donors and recipients is not performed in lung transplantation (LTx). Therefore, whether HLA mismatch between donors and recipients (D/R mismatch) influences postoperative outcomes after LTx remains uncertain. In this study, we investigated the influence of D/R mismatch on postoperative outcomes after cadaveric LTx (CLT).

**Methods:**

A total of 140 CLT procedures were performed between 2012 and 2020. After excluding 5 recipients with preformed DSA and 1 recipient undergoing re-LTx, 134 recipients were enrolled in this retrospective study. The postoperative outcomes were compared between recipients with higher and lower D/R mismatches.

**Results:**

The median D/R mismatch (A/B/DR loci) was 4.0 (range, 1–6). When dividing these 134 recipients into two groups (H group [D/R mismatch ≥ 5, n = 57] and L group [D/R mismatch ≤ 4, n = 77]), there were no significant differences in the patient backgrounds. The lengths of hospital and intensive care unit stays were similar (p = 0.215 and p = 0.37, respectively). Although the overall survival was not significantly better in the H group than in the L group (p = 0.062), chronic lung allograft dysfunction-free survival was significantly better in the H group than in the L group (p = 0.027). Conversely, there was no significant difference in the cumulative incidence of de novo donor-specific anti-HLA antibodies (dnDSAs) between the two groups (p = 0.716).

**Conclusions:**

No significant difference in dnDSA development was observed between patients with higher and lower D/R HLA mismatches. Given the favorable outcomes in the high HLA mismatch group, CLTs can be performed safely in recipients with high D/R HLA mismatches.

**Supplementary Information:**

The online version contains supplementary material available at 10.1007/s11748-024-02109-8.

## Introduction

Lung transplantation (LTx) has been established as the final treatment option to save the lives of patients with various end-stage pulmonary diseases since the long-term survival after LTx was reported by Toronto group [1]. However, as the 5-year overall survival of LTx is reported to be approximately 55%, which is worse than that of other solid organ transplants [2], improvement of LTx outcomes is necessary. One reason for the poor long-term outcomes after LTx is chronic lung allograft syndrome (CLAD). Factors related to CLAD have been reported to include de novo donor-specific anti-HLA antibodies (dnDSAs) and antibody-mediated rejection, which are also reported have relations to short-term postoperative outcomes after LTx [3–5].

In LTx, HLA matching between donors and recipients is generally not performed. Moreover, whether an HLA mismatch between donors and recipients (D/R mismatch) influences prognosis after LTx remains controversial [6–9]. Previously, we reported that the incidence of dnDSAs in cadaveric lung transplantation (CLT) was significantly higher than that in living-donor lobar lung transplantation (LDLLT) [5] and that more attention should be paid to spousal donations in LDLLT because the incidences of the development of dnDSAs and unilateral CLAD were significantly higher in spousal donations than in non-spousal donations [10]. In this study, we retrospectively analyzed the influence of the D/R mismatch on postoperative outcomes after CLT. As HLA typing of cadaveric donors has been performed at the A, B, and DR loci in Japan [4], the D/R HLA mismatch in this study was calculated for the A, B, and DR loci.

## Materials and methods

**Fig. 1:**
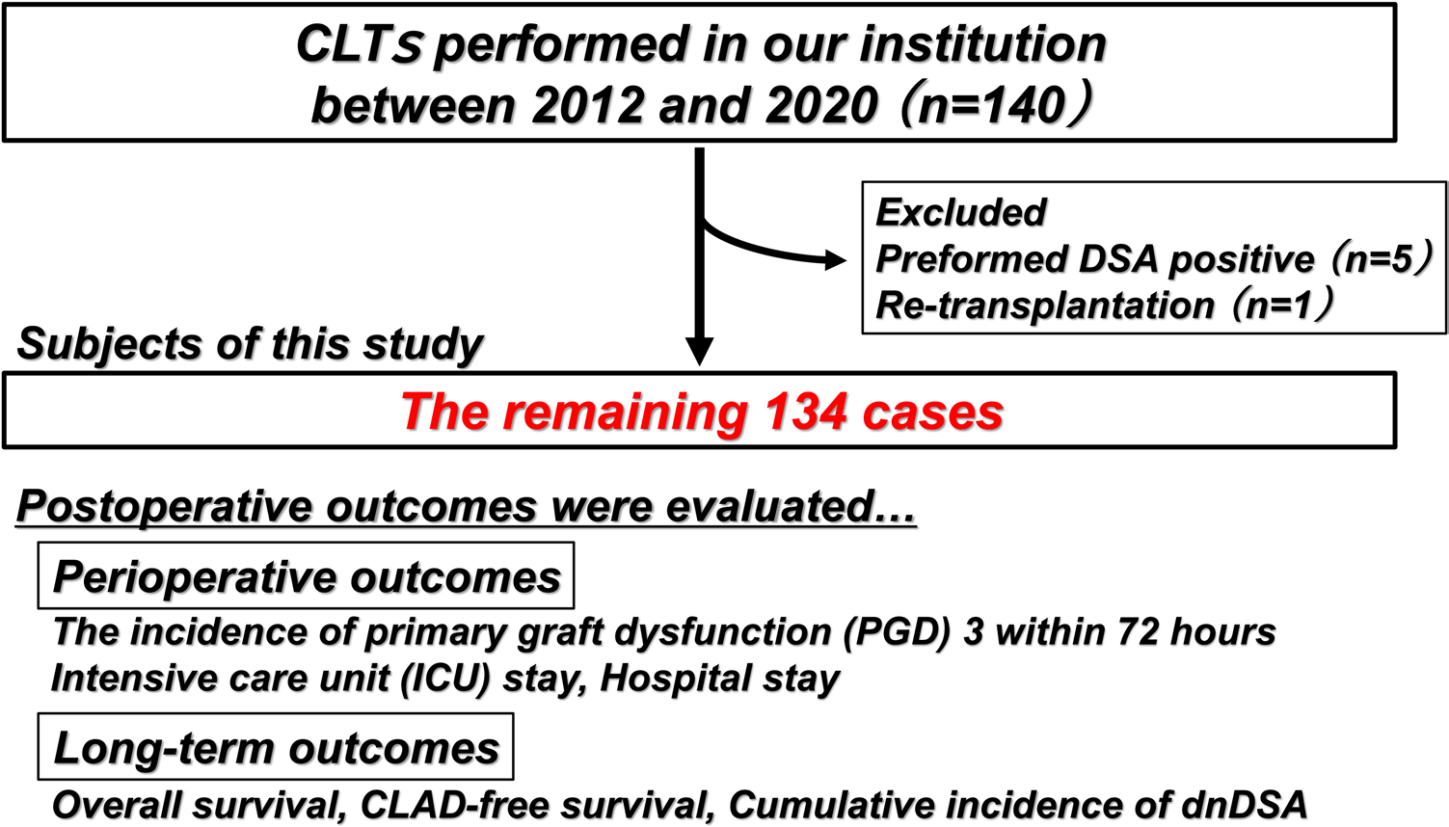
Between 2012 and 2020, 140 cadaveric lung transplantation procedures were performed at our institution. After excluding patients with preformed DSA and those undergoing re-transplantation, the remaining 134 patients were enrolled in this study. Perioperative and long-term outcomes were assessed. *CLT* cadaveric lung transplantation, *DSA* donor-specific antibody, *PGD* primary graft dysfunction, *ICU* intensive care unit, *CLAD* chronic lung allograft dysfunction

Between 2012 and 2020, 140 patients underwent CLT at our hospital. Among them, 134 recipients other than 5 recipients with preformed DSA and one recipient receiving lung re-transplantation, were enrolled in this study (Fig. 1). We analyzed perioperative and long-term postoperative outcomes. Regarding perioperative outcomes, the incidence of primary graft dysfunction (PGD) 3 within 72 h after LTx, intensive care unit (ICU) stay, and hospital stay were evaluated, while overall survival (OS), CLAD-free survival, and cumulative incidence of dnDSA were calculated as long-term outcomes. The follow-up was censored at the end of 2022. This study was approved by the Institutional Review Board (R2389). The requirement for informed consent was waived due to the retrospective nature of this study.

As previously reported [10], periodic screening for anti-HLA antibodies using the LABScreen Mixed kit (One Lambda, CA, USA) has been performed since July 2010. Antibody screening was routinely performed before LTx and one week, one month, three months, six months, and one year after LTx. One year after the LTx, screening was continued annually. Anti-HLA antibodies were also measured when the recipients presented with symptoms or abnormal findings. When the normalized mean fluorescence intensity (MFI) of the anti-HLA antibodies was > 1000, they were considered positive. Antibody specificity was determined using the LABScreen Single Antigen Kit (One Lambda, CA, USA).

The orally administered immunosuppressive agents were calcineurin inhibitors such as cyclosporine or tacrolimus, mycophenolate mofetil, and prednisolone, as previously reported [4, 5, 11].

### Statistical analyses

Descriptive statistics were obtained using the EZR software, a graphical user interface for R (The R Foundation for Statistical Computing, Vienna, Austria) [12]. Continuous variables were presented as medians with ranges, and categorical variables were expressed as percentages. Fisher’s exact test and Mann–Whitney U test were used to compare the two groups. Survival analyses for OS, CLAD-free survival, and incidence of dnDSA were performed using the Kaplan–Meier method, and the groups were compared using a log-rank test. Statistical significance was defined as p < 0.05.

## Results

### Recipient characteristics

The median age was 48 years (range, 4–62 years), and 72 patients (53.7%) were men (Table 1). Sixty-four recipients underwent single CLT, whereas 70 recipients underwent bilateral CLT. The median D/R mismatch was 4.0 (range, 1–6). Based on these results, we divided these 134 recipients into 2 groups: the H group (D/R mismatch ≥ 5, n = 57) and the L group (D/R mismatch ≤ 4, n = 77).

**Table 1:** Patient characteristics and comparison of patient characteristics between patients with higher and lower D/R mismatch

Variables	Total (n = 134)	H group (n = 57)	L group (n = 77)	p value
Age (years)	48 (4–62)	48 (13–62)	48 (4–61)	0.354
Sex				
Male	72 (53.7%)	34 (59.6%)	38 (49.4%)	0.294
Female	62 (46.3%)	23 (40.4%)	39 (50.6%)	
Body mass index (kg/m^2^)	19.1 (10.9–30.5)	19.2 (10.9–30.5)	19.0 (13.8–29.8)	0.709
Indication for CLT				
Interstitial pneumonia	67 (50.0%)	30 (52.6%)	37 (48.1%)	0.81
Idiopathic pulmonary arterial hypertension	15 (11.2%)	6 (10.5%)	9 (11.7%)	
Pulmonary complications after HSCT	12 (9.0%)	6 (10.5%)	6 (7.8%)	
Chronic obstructive pulmonary disease	11 (8.2%)	3 (5.3%)	8 (10.4%)	
Lymphangiomyomatosis	8 (6.0%)	4 (7.0%)	4 (5.2%)	
Bronchiectasis	8 (6.0%)	2 (3.5%)	6 (7.8%)	
Others	13 (9.7%)	6 (10.5%)	7 (9.1%)	
Operative methods				
Single CLT	64 (47.8%)	26 (45.6%)	38 (49.4%)	0.728
Bilateral CLT	70 (52.2%)	31 (54.4%)	39 (50.6%)	
Ischemic time (min)	504.5 (248–780)	504 (274–718)	505 (248–780)	0.597
D/R mismatch (A/B/DR loci)	4.0 (1–6)	5.0 (5–6)	4.0 (1–4)	< 0.001

*CLT* cadaveric lung transplantation; *HSCT*, hematopoietic stem cell transplantation, *D/R mismatch* HLA mismatch between donors and recipients

Between these 2 groups, there were no significant differences in age, sex, or operative methods between the two groups, except for D/R mismatch.

### Comparison of perioperative outcomes

PGD 3 within 72 h after LTx was observed in 19 patients (33.3%) in the H group and 30 patients (39.0%) in the L group (Table 2), indicating that there was no significant difference between the two groups (p = 0.587). The median lengths of ICU and postoperative hospital stay in the H group were 11 days (range, 4–24 days) and 56 days (range, 29–185 days), respectively, which were not significantly different from those in the L group (12 days [range, 3–88 days], p = 0.215 and 63 days [range, 27–440 days], p = 0.37, respectively). Furthermore, the hospital mortality rate was not significantly different between the groups (0% in the H group vs. 3.9% in the L group, p = 0.261).

**Table 2:** Comparison of perioperative outcomes between patients with higher and lower D/R mismatch

Variables	H group (n = 57)	L group (n = 77)	p value
PGD3 within 72 h (%)	19 (33.3%)	30 (39.0%)	0.587
ICU stay (days)	11 (4–24)	12 (3–88)	0.215
Hospital stay (days)	56 (29–185)	63 (27–440)	0.37
Mortality (%)	0 (0%)	3 (3.9%)	0.261

*D/R mismatch* HLA mismatch between donors and recipients, *PGD* primary graft dysfunction, *ICU* intensive care unit

### Comparison of long-term outcomes

**Fig. 2:**
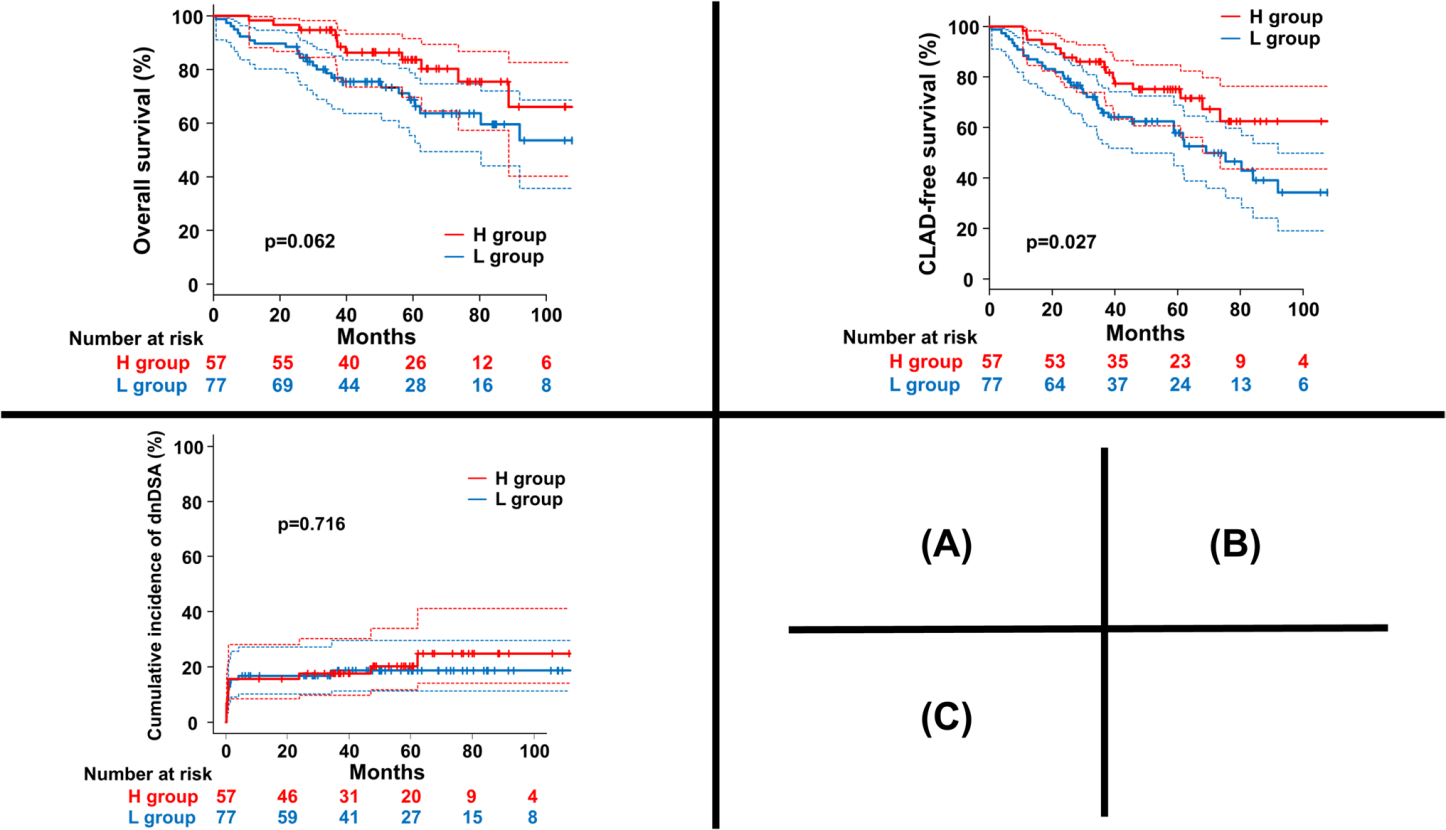
**A** Although the difference was not significant, overall survival (OS) in the H group tended to be better than that in the L group. Five-year OS in the H group was 83.6% (95% confidence interval [CI]: 69.8–91.6%), while that in the L group was 68.7% (95% CI: 55.5–78.7%). **B** There was a significant difference in CLAD-free survival between the H group and the L group (p = 0.027). CLAD-free survival in the H group was 75.0% (95% CI: 60.7–84.8%]) and that in the L group was 57.9% (95% CI: 44.8–68.9%). (C) There was no significant difference in the cumulative incidence of de novo donor-specific antibody (dnDSA). Five-year cumulative incidences of dnDSA in the H group and the L group were 20.4% (95% CI: 11.7–34.1%), and 18.7% (95% CI: 11.5–29.5%), respectively. *OS* overall survival, *CI* confidence interval, *CLAD* chronic lung allograft dysfunction, *dnDSA *de novo donor-specific anti-HLA antibody

The OS of the H group was better than that of the L group, although the difference was not significant (P = 0.062). Five-year OS in the H group was 83.6% (95% confidence interval [CI]: 69.8–91.6%), and that int the L group was 68.7% (95% CI: 55.5–78.7%, Fig. 2A). Furthermore, five-year CLAD-free survival in the H group was significantly better than that in the L group (H group: 75.0% [95% CI: 60.7–84.8%] and L group: 57.9% [95% CI: 44.8–68.9%], p = 0.027, Fig. 2B). However, no significant difference in the cumulative incidence of dnDSA was observed between the 2 groups (p = 0.716). Five-year cumulative incidence of dnDSA in the H group was 20.4% (95% CI: 11.7–34.1%) and that in the L group was 18.7% (95% CI: 11.5–29.5%, Fig. 2C). The median duration between the appearance of dnDSA and CLT in the H group was 22.5 days (range, 8–1893 days), while that in the L group was 25 days (range, 5–1044 days).

For reference, we performed a supplemental analysis in which the recipients were divided into two groups using another cut-off line (D/R mismatch ≥ 4 [H’ group, n = 101] and D/R mismatch ≤ 3 [L’ group, n = 33]). There was no significant difference in OS between these two groups (p = 0.695, Supplemental Fig. 1A). Five-year OS in the H’ group was 75.9% (95% CI: 65.5–83.6%), while that in the L’ group was 73.0% (95% CI: 50.3–86.5%). Similarly, the CLAD-free survival was similar (5-year CLAD-free survival: 65.4% [95% CI: 54.5–74.4%] in the H’ group vs 64.7% [95% CI: 43.5–79.6%] in the L’ group, p = 0.645, Supplemental Fig. 1B). The cumulative incidence of dnDSA in the H’ group was slightly higher than that in the L’ group; however, the difference was not significant (p = 0.221). The five-year incidence in the H’ group was 21.5% (95% CI: 14.5–31.2%), and that in the L’ group was 13.4% (95% CI: 5.2–32.4%).

During the observation period, 36 patients died, and 33 patients developed CLAD. Among 35 of these 36 patients, after excluding one patient who died within 90 days, the most frequent cause of death was CLAD (n = 16), followed by infection (n = 9) and malignancy (n = 6).

### Subgroup analysis – analysis among female recipients

For subgroup analyses, further investigations on the outcomes of CLT in female recipients were performed, as pre-sensitization through pregnancy and delivery is sometimes observed among female recipients. Of the 62 female recipients, 35 had a history of pregnancy. Recipients with a history of pregnancy were significantly older than those without a history of pregnancy (median age: 49 vs. 35 years, p < 0.001) and received a single CLT more frequently (45.7% vs. 14.8%, p = 0.014, Supplemental Table 1).

Regarding the perioperative outcomes, there were no significant differences in PGD 3 within 72 h after CLT, the lengths of ICU stay and hospital stay (p = 0.798, p = 0.994, and p = 0.088, respectively; Supplemental Table 2). Furthermore, there were no significant differences in the long-term outcomes. Five-year OS among recipients with history of pregnancy was 78.8% (95% CI: 57.4–90.3%), whereas that among recipients without history of pregnancy was 88.0% (95% CI: 66.9–96.0%, p = 0.468, Supplemental Fig. 2A). Five-year CLAD free survival was 64.9% (95% CI: 43.4–79.9%) among patients with history of pregnancy, and 78.8% (95% CI: 55.6–90.8%) among patients without history of pregnancy (p = 0.126, Supplemental Fig. 2B). Furthermore, although the 5-year cumulative incidence of dnDSA among patients with a history of pregnancy was higher than that among patients without a history of pregnancy, the difference was not significant (31.4% [95% CI: 18.8–49.5%] vs. 11.1% [95% CI: 3.7–30.6%], p = 0.093, Supplemental Fig. 2C).

## Discussion

Several important findings were observed in this study. No significant differences in perioperative outcomes or the cumulative incidence of dnDSA development were observed between patients with higher and lower D/R mismatches. Although not significant, patients with a higher D/R mismatch tended to have a better OS than those with a lower D/R mismatch, whereas the CLAD-free survival in cases with a higher D/R mismatch was significantly better than that in cases with a lower D/R mismatch. Based on these findings, CLTs can be performed safely in recipients with high D/R HLA mismatch.

In this analysis, the median D/R mismatch in the A, B, and DR loci was 4.0, which is comparable to a previous report from another institution [13]. However, there was no significant difference in the cumulative incidence of dnDSA between the H and L groups, and the median duration between dnDSA development and CLT was approximately 25 days in both groups, indicating that dnDSA development in CLT occurred early after CLT, regardless of the extent of D/R mismatch. This result was quite different from that obtained in a previous study on LDLLT, which showed that the median duration between dnDSA development and LDLLT was 758 days for spousal LDLLT and 307 days for non-spousal LDLLT [10]. In contrast, the 5-year cumulative incidence of dnDSA in this study was 20.4% in the H group and 18.7% in the L group, which is comparable to the results from other institutions in Japan that reported a dnDSA occurrence of 16% [14], suggesting that the results in this study were reasonable.

There were no significant differences in patient backgrounds between patients with lower and higher D/R mismatches, and no significant differences in perioperative outcomes between the two groups were observed. However, regarding long-term outcomes, OS in patients with a higher D/R mismatch tended to be better than that in patients with a lower D/R mismatch, although not significant. Furthermore, CLAD-free survival in patients with a higher D/R mismatch was significantly better than that in patients with a lower D/R mismatch. Previously, OS in patients with higher D/R mismatch was reported to be worse than that in patients with lower D/R mismatch [6, 9], and CLAD development was observed more frequently in patients with higher D/R mismatch than that in those with lower D/R mismatch [7, 9, 15]; however, there has been no report on the better outcomes of patients with higher D/R mismatch. The reasons for the results of this study remain uncertain, but some factors can be considered as potential influencers. CLAD can be induced by various factors including airway infections such as pneumonia. The prevention and control of infection are sometimes difficult among lung transplant recipients, since immunosuppression is usually stronger than other solid organ transplants. Previously, we reported that the single nucleotide polymorphism (SNP) in Fc gamma receptor IIA was related to the occurrence of infectious complications after lung transplantation [16]. Such genetic factors possibly influencing postoperative outcomes after lung transplantation including SNPs might have affected the results of this study. Moreover, the report from Japanese lung transplant registry report indicated that recipient age, indication for lung transplantation, and gender mismatch between donors and recipients had influences on prognoses after lung transplantation [17]. In this study, although there was no significant difference in patient backgrounds between the H group and the L group, it cannot be denied that these factors have possibly influenced on post-LTx outcomes. On the other hand, there are some reports on native lung complications after single CLT [18–20]. We considered it as a possible influencing factor; however, since the postoperative outcomes after single CLT have been reported acceptable, and since about the half of the patients in the both groups underwent single CLT in the present study, they were unlikely to be related to the postoperative outcomes. To validate the results of this study, a larger scale of study in the future is desired, but it is no exaggeration to say that given the favorable outcomes in the higher HLA mismatch group, CLTs for recipients with high D/R HLA mismatch can be safely performed.

In comparison with other solid organ transplants, D/R mismatch has a significant influence on prognosis among adult living-donor liver transplant recipients [21], whereas D/R mismatch does not affect postoperative outcomes in cadaveric liver transplantation [22]. These reports are consistent with the results of living and cadaveric lung transplantations at our institution. In this study, detailed analyses of the influence of each locus on the postoperative outcomes were not performed because of the limited number of patients. As future perspectives, since the HLA mismatch status of the DR locus was reported to have some influence on graft survival [8], we will continue this research and consider reporting the results in the near future.

We performed subgroup analyses of female recipients to investigate the influence of pre-sensitization due to pregnancy and delivery. The OS and CLAD-free survival rates were not significantly different between female recipients with and without a history of pregnancy. The cumulative incidence of dnDSA was higher in recipients with a history of pregnancy; however, this difference was not significant. Based on these results, it was concluded that we need not pay too much attention to pregnant history among female recipients of CLT, although we should consider that some factors of patient backgrounds were significantly different between the two groups, and that, since pre-sensitization can occasionally lead to positive complement-dependent cytotoxicity cross-match, pre-sensitized female recipients who can undergo CLT might have been biased.

This study has some limitations. First, this was a single-center, retrospective, non-randomized study. Therefore, multicenter studies with larger cohorts are required to validate our findings. Second, owing to the allocation system in Japan, D/R mismatch was calculated only for the A, B, and DR loci. As some studies have shown that D/R mismatch of the DQ locus is also important [23, 24], it is desirable to add HLA typing of the DQ locus in the near future.

In summary, although no significant differences in overall survival and cumulative incidence of dnDSA development were observed between patients with higher and lower D/R mismatches, CLAD-free survival in the higher D/R mismatch group was significantly better than that in the lower D/R mismatch group. Based on the favorable outcomes in the high D/R mismatch group, CLTs can be performed safely in recipients with high HLA mismatches.

## Supplementary Information

Below is the link to the electronic supplementary material.

Supplementary file1 (TIF 26385 KB)

Supplementary file2 (TIF 26385 KB)

Supplementary file3 (DOCX 19 KB)

## Data Availability

The data analyzed in this study are available from the corresponding author on reasonable request.

## Abbreviations

LTx: Lung transplantation
CLAD: Chronic lung allograft dysfunction
dnDSA: De novo Donor-specific anti-HLA antibody
D/R mismatch: HLA mismatch between donors and recipients
CLT: Cadaveric lung transplantation
LDLLT: Living-donor lobar lung transplantation
PGD: Primary graft dysfunction
ICU: Intensive care unit
OS: Overall survival
MFI: Mean fluorescence intensity
CI: Confidence interval
