# Supplementary figures and images for: Influence of HLA mismatch between donors and recipients on postoperative outcomes in cadaveric lung transplantation
Source: Gen Thorac Cardiovasc Surg. 2024 Dec 9;73(8):609–15. doi: 10.1007/s11748-024-02109-8 (PMC12289801; doi:10.1007/s11748-024-02109-8)

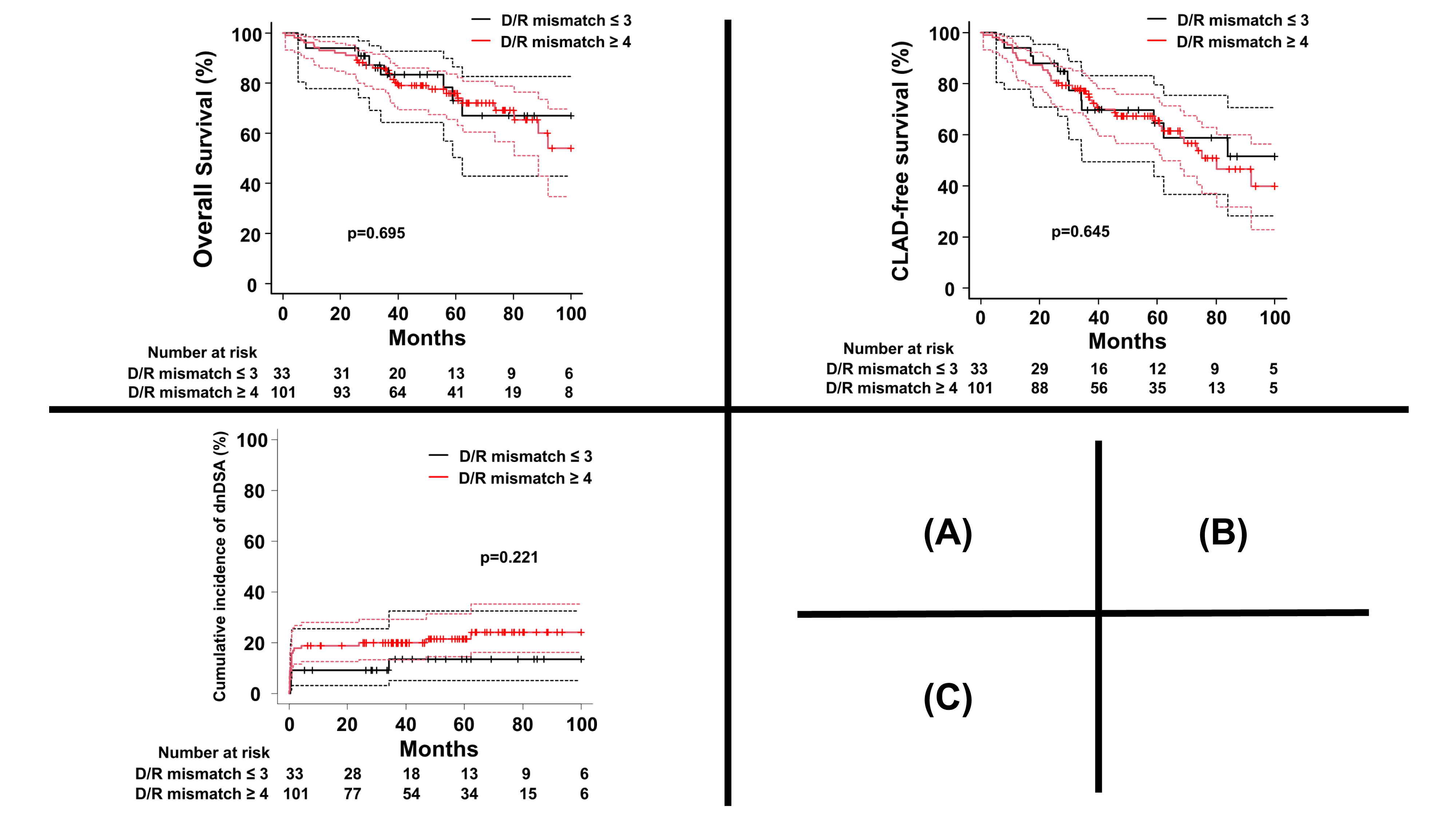

Supplement: Supplementary file 1 — Supplementary file1 (TIF 26385 KB) [file 11748_2024_2109_MOESM1_ESM.tif]

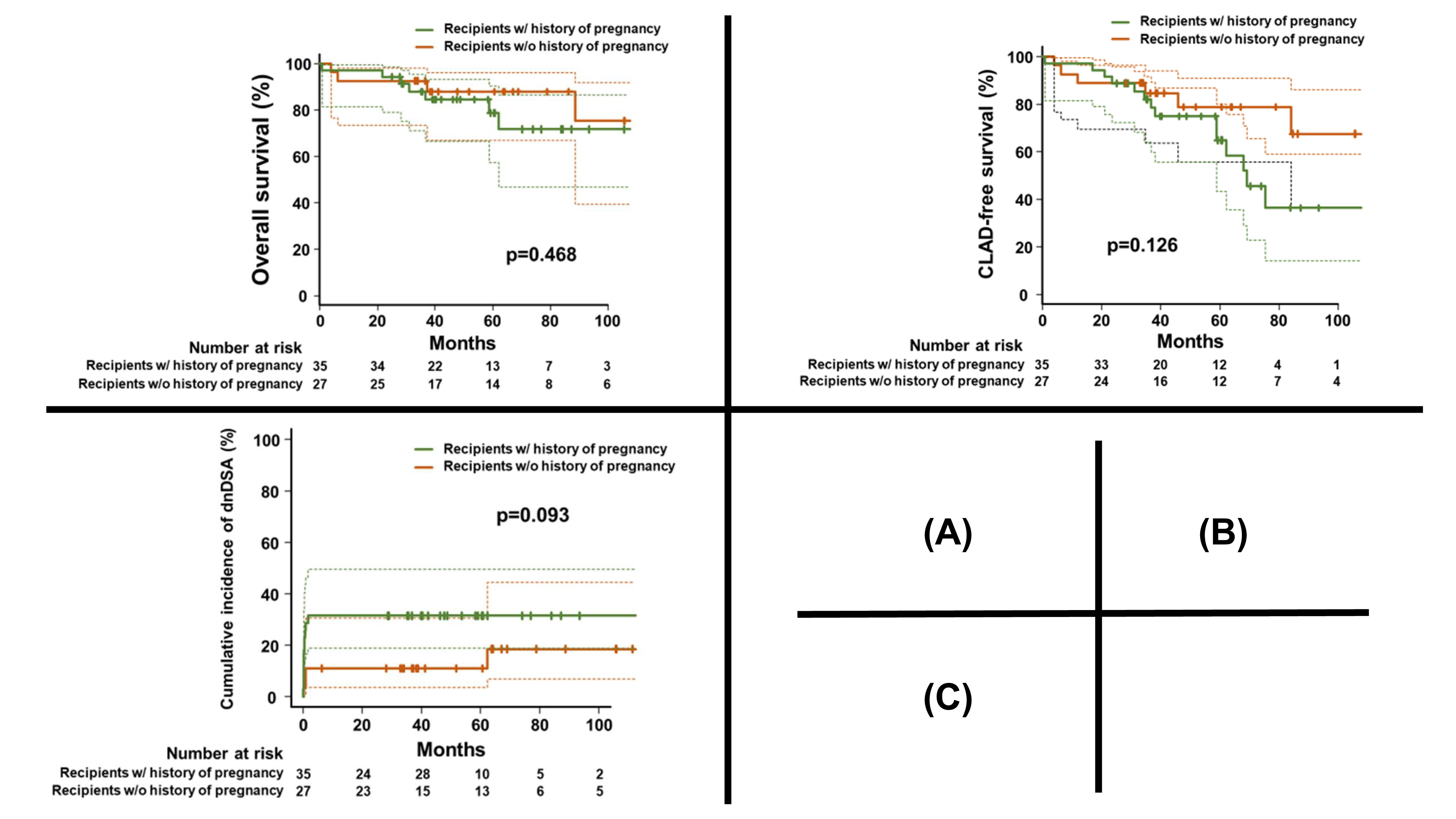

Supplement: Supplementary file 2 — Supplementary file2 (TIF 26385 KB) [file 11748_2024_2109_MOESM2_ESM.tif]
