# Supplementary material for: Influence of HLA mismatch between donors and recipients on postoperative outcomes in cadaveric lung transplantation
Source: Gen Thorac Cardiovasc Surg. 2024 Dec 9;73(8):609–15. doi: 10.1007/s11748-024-02109-8 (PMC12289801; doi:10.1007/s11748-024-02109-8)
Supplement: Supplementary file 3 — Supplementary file3 (DOCX 19 KB) [file 11748_2024_2109_MOESM3_ESM.docx]

**Supplemental Table 1.**

Comparison of patient characteristics between patients with and without history of pregnancy.

| Variables | Patients with history of pregnancy (n=35) | Patients without history of pregnancy (n=27) | p value |
| --- | --- | --- | --- |
| Age (years) | 49 (28-61) | 35 (4-59) | <0.001 |
| Body mass index (kg/㎡) | 20.1 (15.2-29.8) | 16.8 (10.9-21.8) | <0.001 |
| Indication for CLT |  |  |  |
| - Interstitial pneumonia | 18 (51.4%) | 6 (22.2%) | 0.035 |
| - Idiopathic pulmonary arterial hypertension | 5 (14.3%) | 7 (25.9%) |  |
| - Pulmonary complications after HSCT | 0 (0.0%) | 3 (11.1%) |  |
| - Chronic obstructive pulmonary disease | 2 (5.7%) | 0 (0.0%) |  |
| - Lymphangiomyomatosis | 6 (17.1%) | 2 (7.4%) |  |
| - Bronchiectasis | 2 (5.7%) | 3 (11.1%) |  |
| - Others | 2 (5.7%) | 6 (22.2%) |  |
| Operative methods |  |  |  |
| - Single CLT | 16 ( 45.7) | 4 ( 14.8) | 0.014 |
| - Bilateral CLT | 19 ( 54.3) | 23 ( 85.2) |  |
| D/R mismatch (A/B/DR loci) | 4.0 (1-6) | 5.0 (2-6) | 0.032 |

*Abbreviations*

CLT, cadaveric lung transplantation; HSCT, hematopoietic stem cell transplantation; D/R mismatch, HLA mismatch between donors and recipients.

**Supplemental Table 2.**

Comparison of perioperative outcomes between patients with and without history of pregnancy.

| Variables | Patients with history of pregnancy (n=35) | Patients without history of pregnancy (n=27) | p value |
| --- | --- | --- | --- |
| PGD3 within 72 hours (%) | 16 (45.7%) | 11 (40.7%) | 0.798 |
| ICU stay (days) | 14 (5-57) | 16 (6-88) | 0.994 |
| Postoperative hospital stay (days) | 60 (27-420) | 66 (38-192) | 0.088 |
| Mortality (%) | 1 (2.9%) | 2 (7.4%) | 0.575 |

*Abbreviations*

D/R mismatch, HLA mismatch between donors and recipients; PGD, primary graft dysfunction; ICU, intensive care unit.
